# Understanding How Infrared Beak Treatment Affects the Beak Tissue and the Healing Response of Brown and White Feathered Layer Pullets

**DOI:** 10.3390/ani9090665

**Published:** 2019-09-07

**Authors:** Sarah Struthers, Ashish Gupta, Susantha Gomis, Eugenia Herwig, Karen Schwean-Lardner

**Affiliations:** 1 Department of Animal and Poultry Science, University of Saskatchewan, Saskatoon, SK S7N 5A8, Canada (S.S.) (E.H.); 2 Department of Veterinary Pathology, Western College of Veterinary Medicine, University of Saskatchewan, Saskatoon, SK S7N 5B4, Canada (A.G.) (S.G.)

**Keywords:** histology, beak treatment, beak length, animal welfare

## Abstract

**Simple Summary:**

Beak treatment of commercial laying hens remains an important management practice as it is one of the most effective methods of controlling and preventing cannibalism. Infrared beak treatment is the most recent beak treatment method to be utilized and the available literature shows that it has less of a negative impact on birds compared to older methods of beak treatment. Although there is considerable research evaluating the impact of infrared beak treatment on the production and welfare of laying hens, it is still not fully understood how it affects the beak tissue during the first few days post treatment. This is important to understand as it can provide insight into whether or not treated birds are experiencing pain, which has consequences for both welfare and productivity. This study examined the effect of infrared beak treatment on the histology of the beak during early life (first 21 days). Epithelial regeneration started as soon as five days post treatment. There was no evidence that infrared beak treatment resulted in the formation of neuromas or any other indication of chronic pain.

**Abstract:**

Infrared beak treatment has less of a negative impact on laying hen welfare compared to other methods of beak treatment; however, it is still not fully understood how infrared beak treatment affects the beak tissue during the first few days post treatment. The objective of this study was to examine the histology of infrared beak treated vs. untreated beaks of 2 strains of layer chicks during early life. One-hundred Lohmann Brown-Lite (LB) and 100 Lohmann LSL-Lite (LW) chicks were obtained; 50 chicks per strain were infrared beak treated post hatch (IR) with the remainder being sham untreated controls (C). Data collected included presence of beak sloughing, length, and histology. Histology slides were analyzed and scored on a scale of 0 to 4, with 0 indicating no lesions and 4 indicating severe inflammation. Sloughing of the treated beak tissue began at 10 days and was complete by 20 days. IR pullets had shorter beak lengths once sloughing was initiated and less overall beak growth. No differences in healing scores were found between treated LB and LW beaks; all treated LB beaks were healed by 21 days while some LW beaks still showed inflammation. Overall, infrared beak treatment was effective at reducing beak growth post treatment. Healing occurred post treatment in both strains as evident by complete regeneration of the epithelium and a reduction in inflammation.

## 1. Introduction

In commercial egg production, hens are beak treated to control cannibalism and feather pecking. Traditional methods of beak treatment, such as hot-blade trimming, involve the use of a heated blade to cut and cauterize the beak tissue [1]. Hot-blade trimming results in acute pain and, depending on the age at trimming and the severity of the trim, may cause chronic pain and the formation of neuromas (proliferative masses of nerves that develop at the end of severed nerves) [2,3,4]. A more recent method, infrared beak treatment, uses a non-contact, high intensity infrared light, which penetrates the keratinized outer layer of the beak (rhamphotheca) and damages the underlying tissue layers, stopping further regeneration of the beak tissue [5]. The treated beak tissue gradually sloughs off in 1 to 2 weeks post treatment, allowing the bird time to adapt to the change in beak shape. 

As with many other animal husbandry practices, beak treatment often raises concerns (valid or not) regarding its effect on welfare. Various alternatives to beak treatment, such as varying light intensity, using low feather pecking strains, and the use of enrichments, have been proposed and studied [1]. However, very few of these alternatives are currently as effective as beak treatment. The eventual goal is that beak treatment will no longer need to be practiced. However, until reliable, effective alternatives are found, it is important to study and understand the effects of beak treatment (specifically infrared beak treatment) on the productivity and welfare of different poultry species. 

The majority of the scientific literature on welfare and beak treatment has focused on hot-blade trimming, and often these results are extrapolated to what might occur with the use of infrared beak treatment. However, previous studies comparing hot-blade trimming and infrared beak treatment have found that infrared beak treatment represents a more welfare-friendly method of beak treatment because it has less of a negative effect on pullet and hen production and welfare [6,7,8]. Infrared beak treatment has been found to cause fewer behavioural changes, improve feather cover, reduce aggression, and limit tissue damage that could cause acute and/or chronic pain [6,8]. Additionally, research is available demonstrating the effect of hot-blade trimming on beak anatomy and healing, but similar studies are lacking for infrared beak treatment, particularly during the first few days post treatment. Age at trimming is an important factor for healing and neuroma formation post beak treatment, particularly with hot-blade trimming [8]. When birds are treated within a few days post-hatch using hot-blade trimming, healing is more rapid compared to birds trimmed at older ages; however, neuromas were not observed post treatment at any of the trimming ages [3,9]. 

There is still limited research investigating the long-term effects of infrared beak treatment on the neurophysiology and anatomy of the beak. Chicks that were treated using infrared beak treatment at 1 day of age were found to have neuromas, which were observed at 32 days of age and persisted into adulthood [10]. Neuroma formation occurred because a more severe treatment setting was used, causing excessive beak tissue to be removed [11]. Following the work conducted by Glatz and Hinch [10], less severe treatment settings are being used on laying hens worldwide to help prevent neuroma formation. McKeegan and Philbey [11] found that infrared beak treatment on day of hatch did not have an effect on nociceptor (pain) thresholds in laying hens at 10, 30, and 50 weeks of age, suggesting that infrared beak treatment does not result in chronic pain. Treated beaks showed no evidence of pathological changes to the beak structure and underwent appropriate healing, as evident by re-epithelialisation and nerve regeneration. Although the authors demonstrated that infrared beak treatment did not negatively affect the sensory function of the beak, histology was not conducted until birds were 4 weeks old. As such, the healing process in the days immediately following infrared beak treatment is still not fully understood. 

Despite the research that has been conducted on the impact of infrared beak treatment on the production and welfare of egg production pullets, gaps in the scientific literature regarding the effects of infrared beak treatment on beak histology and healing still exist. Therefore, the objectives of this study were to understand the histology of infrared beak treated versus untreated beaks during early life and investigate how infrared beak treatment affects the beak length and healing response of Lohmann Brown-Lite (LB) and Lohmann LSL-Lite (LW) pullets. Two egg-layer strains were used in the present study to determine how different genotypes are affected by infrared beak treatment. 

## 2. Materials and Methods 

This work was approved by the University of Saskatchewan’s Animal Research Ethics Board (AUP 19940248) and adhered to the Canadian Council on Animal Care guidelines for humane animal use [12].

Newly hatched LB and LW female chicks (*n* = 100 per strain) were obtained from a commercial hatchery. Prior to arrival at the research facility, 50 chicks per strain were infrared beak treated immediately post hatch (IR) by a trained operator. The remaining chicks were sham untreated controls (C), meaning that they were handled and placed into the infrared beak treatment equipment, but their beaks were not exposed to the infrared light. Infrared treatment settings for each strain are described in Table 1. The guard-plate determines how much of the top beak is exposed to the infrared light; the mirror design (shape and material) determines how much light is reflected back onto the bottom beak; and the power determines how deep the infrared light penetrates the beak tissue. Beak exposure time was approximately 1.5 s. Different power settings were used for the LW and LB strains because of factors, such as bird strain, flock age, production environment, and genetic differences in beak pigment, hardness, and shape. At the research facility, chicks (*n* = 44 per treatment) were housed in experimental cages (*n* = 44) from 0 to 21 days of age with 4 chicks per cage (625 cm^2^ per chick). Research staff were not blinded to which treatments the chicks belonged to. All pullets had ad libitum access to commercial chick starter and water. Feed was provided via chick feeders for the first 14 days and then in trough feeders for the remaining time. Water was provided through 360° nipple drinkers (2 nipples per cage), with supplemental waterers provided for the first 14 days. The photoperiod was 23L:1D (20 lux) for the first 7 days and then 8L:16D (15 lux) from day 8 onwards using incandescent light bulbs as the light source and included dawn and dusk periods of 15 minutes each. Heat was provided via hot water pipes running along the walls of the room. Room temperature started at 32 °C at 1 day and decreased by approximately 2 °C each week to reach a final room temperature of 25 °C at 21 days of age.

Starting at 7 days of age, all IR pullets were examined daily to determine initiation and completion of beak sloughing. To perform this assessment, pullets were removed from their cages one at a time and their top and bottom beaks were examined by a trained research technician and identified as either intact, partially sloughed, or completely sloughed. 

**Figure 1:**
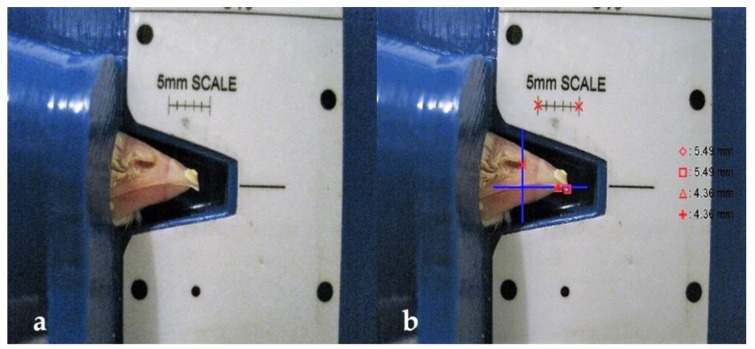
(**a**) Nova-Tech Engineering LLC beak scale; (**b**)calculation of beak length using ImageJ software.

At 1, 7, 14, and 21 days of age, digital photographs were taken of the beaks of all pullets using the Nova-Tech Engineering LLC beak scale (Figure 1a) and a Canon PowerShot SD1200IS camera (Canon Canada Inc., Mississauga, ON, Canada). Photographs were analyzed to calculate beak length (distance between the anterior end of the nares to the end of the upper and lower beak at each age) and overall beak growth (difference in beak length at 21 and 1 day of age) using ImageJ analysis software (version 1.52, National Institutes of Health, Bethesda, MD, USA) (Figure 1b). 

Beak samples were collected for histology from 4 pullets per treatment every 2 days starting at 1 day of age. Pullets were humanely euthanized using manual cervical dislocation and their beaks were removed by cutting where the beak attached to the skull. Beaks were then placed in 10% neutral buffered formalin and stored at room temperature for a minimum of 2 days prior to trimming. The beaks were gross trimmed into sagittal cross sections of approximately 5 mm and placed in cassettes. Samples were then submitted to an independent diagnostic laboratory for slide preparation (decalcified for 15 h in 20% formic acid, embedded in paraffin wax, sectioned at 5 µm, and stained with hematoxylin and eosin (H&E) (SelecTech Hematoxylin 560 and SelecTech Alcoholic Eosin Y515, Leica Biosystems, Winnipeg, MB, Canada)). Using the beak healing classifications described in Table 2, slides were examined by a veterinary pathologist and scored on a scale of 0 to 4, with 0 showing no lesions and 4 showing severe inflammation and necrosis. Due to difficulty in obtaining complete sections of the bottom beak, only slides containing top beaks were scored. To avoid bias, the pathologist was blind to which slides belonged to which treatment and all slides were analyzed at the same time.

The experiment was designed as a 2 × 2 factorial arrangement of beak treatment and strain, in a completely randomized design with 44 replicates per treatment (bird as replicate unit). Beak length data were analyzed using PROC MIXED (SAS 9.4, Cary, NC, USA) with Tukey’s range test to separate means. The non-parametric ordinal histological score data (infrared treated beaks only) were analyzed as a one-way analysis of variance of strain using PROC NPAR1WAY (SAS 9.4, Cary, NC, USA) and the Kruskal–Wallis test, with 88 replicates per strain. Differences were considered significant when *p* ≤ 0.05 and a trend was noted when 0.05 < *p* ≤ 0.10.

## 3. Results

### 3.1. Beak Sloughing

**Figure 2:**
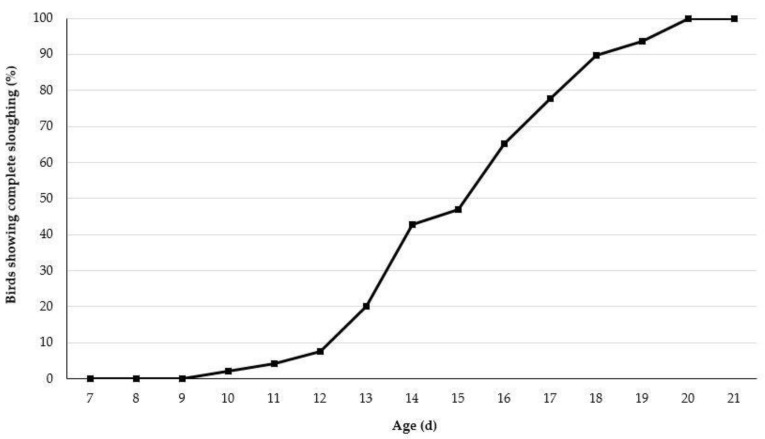
Percentage of infrared beak treated pullets showing complete (top and bottom) beak sloughing from 7 to 21 days of age.

Sloughing of the treated beak tissue was initiated at 10 days of age in approximately 2 percent of pullets and was complete by 20 days of age (Figure 2). For both strains, the sloughing process began at 10 days of age. For LW pullets, it was complete by 17 days of age and for LB pullets, it was complete by 20 days.

### 3.2. Beak Length and Growth

Infrared beak treatment was effective at reducing beak length and growth post treatment. There was a trend for C pullets to have longer top beaks than IR pullets at 1 day of age (*p* = 0.096). At 21 days of age, C pullets also had longer top beak lengths compared to IR pullets (9.91 mm vs. 6.06 mm, respectively). The strains demonstrated differences in top beak length with LB pullets having longer top beaks than LW at 1 day of age, but shorter top beaks at 7 days of age (Table 3). No differences in top beak length were observed between LB and LW pullets after 7 days. An interaction between beak treatment and strain was noted in top beak length at 14 days of age with untreated LB and LW pullets having longer top beaks (8.49 mm and 8.58 mm, respectively) compared to treated LB and LW pullets (7.36 mm and 6.47 mm, respectively). Within the IR treatment, LB pullets had longer top beaks than LW pullets (7.36 mm vs. 6.47 mm, respectively). Bottom beak length was affected by beak treatment at every age with IR pullets having longer bottom beaks than C pullets at 1 day and 7 days of age but shorter bottom beaks at 21 days. At 1 day of age, LB pullets had longer bottom beaks compared to LW (4.79 mm vs. 4.53 mm, respectively). 

Over the 21-day period, C pullets had more beak growth compared to IR pullets (4.38 mm vs. 0.63 mm for top beak; 3.88 vs. 0.76 mm for bottom beak). LB pullets had less overall beak growth compared to LW pullets over the 21 days (2.35 mm vs. 2.66 mm for top beak; 2.15 mm vs. 2.51 mm for bottom beak). 

### 3.3. Beak Histology

**Figure 3:**
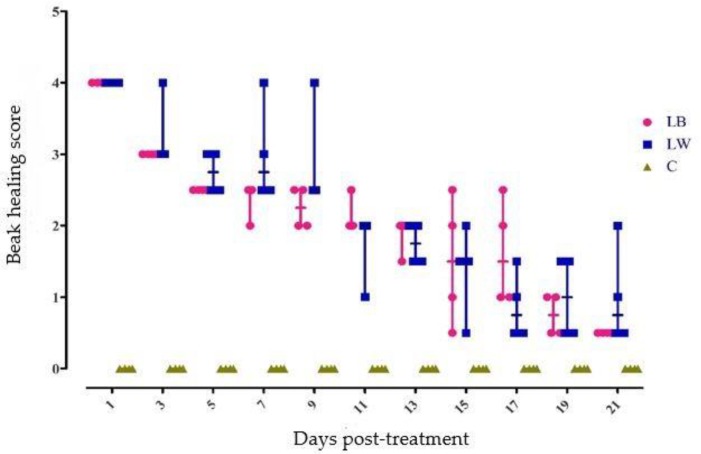
Beak healing scores for infrared beak treated Lohmann Brown-Lite (LB) and Lohmann LSL-Lite (LW) pullets compared to sham treated control (C) pullets (scores identical for C pullets from each strain). Descriptions for histological scores are provided in Table 2. Each marker represents a single bird. Dashes on the lines represent the median score.

**Figure 4:**
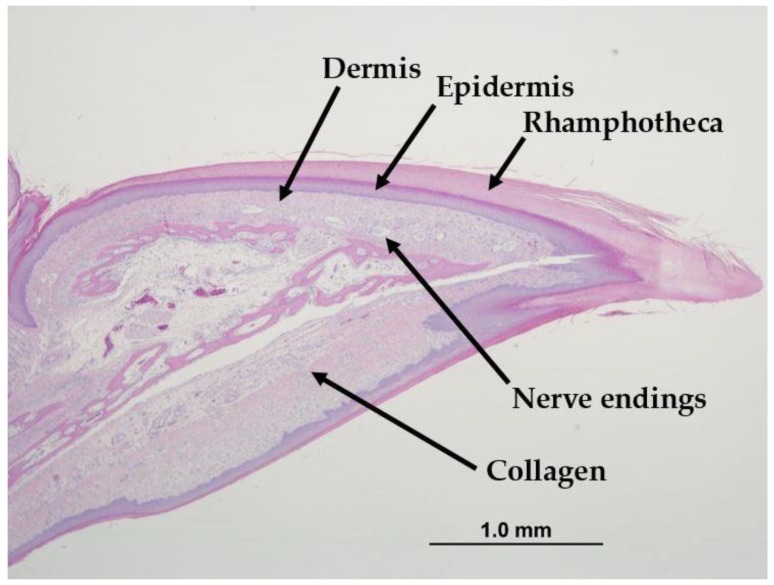
Histological section of the top beak of a C pullet. The three tissue layers of the beak (rhamphotheca, epidermis, and dermis) are indicated. Multiple nerve endings (Herbst corpuscles) and mature collagen bundles are visible within the dermis layer. H&E staining. Magnification 4×.

Figure 3 shows the beak healing scores for treated LB and LW top beaks every 2 days from 1 day to 21 days of age. The scores were based on a scale from 0.5 to 4, with a 0 given only for untreated control beaks. There were no significant differences in beak healing scores between treated LB and LW beaks at any age. Figure 4 shows the anatomy of a normal, untreated beak. The three tissue layers of the beak are present: the rhamphotheca (outer keratinized layer), the epidermis (consisting of stratified squamous epithelial cells), and the dermis (consisting of dense collagen fibres and contains nerve endings and blood vessels). 

**Figure 5:**
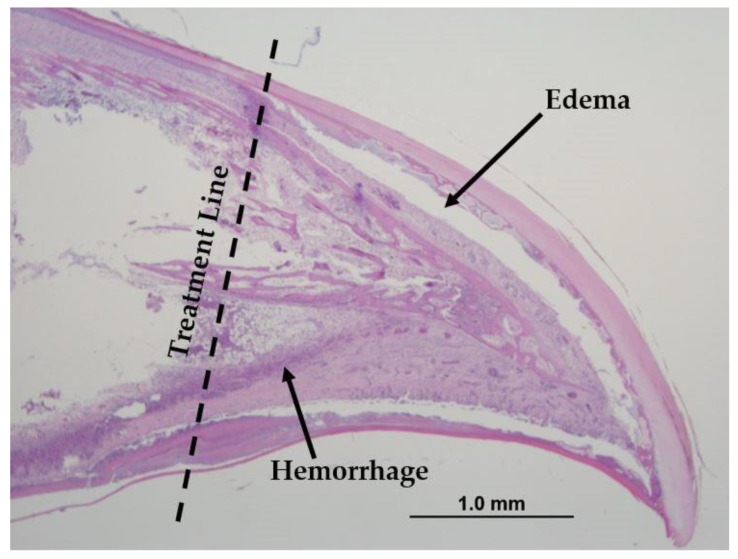
Histological section of the top beak of an IR pullet at 1 day post treatment (score of 4). The beak tissue below the treatment line is dead (coagulative necrosis) but still intact. Cellular infiltration, edema, and hemorrhage are present. H&E staining. Magnification 4×.

**Figure 6:**
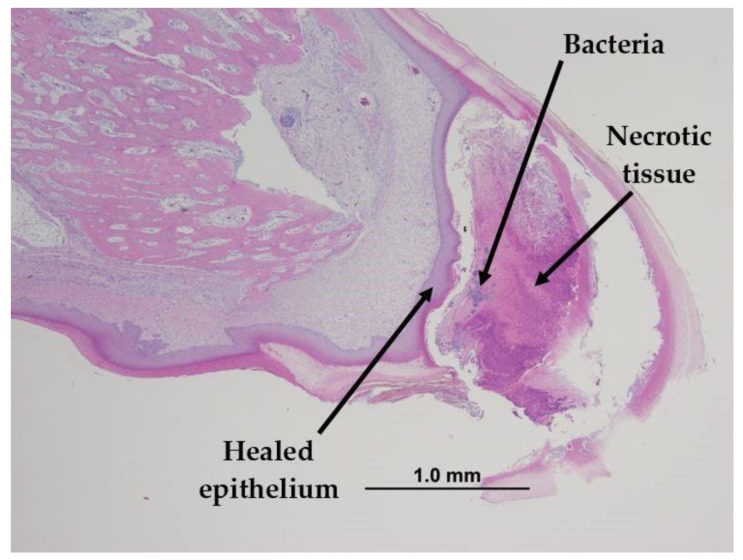
Histological section of the top beak of an IR pullet at 17 days post treatment. The beak is completely healed as evident by the united epithelial layer. Although the beak is healed, the necrotic beak tip has not sloughed. Bacteria is present within the necrotic tissue only. H&E staining. Magnification 4×.

At 1 day post treatment, all treated beaks, regardless of strain, showed coagulative necrosis of the beak tissue below the treatment line (Figure 5). Cellular infiltration, edema, and hemorrhage were also observed. Regeneration of the epithelial layer (epidermis) was visible in both strains at 5 days post treatment, and by 9 days post treatment, bone healing and the formation of new blood vessels were occurring. At 17 days post treatment, the epithelial layer was completely regenerated and covered the entire beak tip; however, the necrotic tissue still had not sloughed (Figure 6). Bacteria was present within the necrotic tissue but, more importantly, it was not present within the healed tissue. All treated LB beaks showed complete healing at 21 days of age, while one LW beak stilled showed only moderate healing. Infrared beak treatment did not result in neuroma formation or cause abnormal nerve growth post treatment in any of the beaks sampled over the 21-day period. 

## 4. Discussion

Infrared beak treatment had a quick impact on the beak tissue, as a trend appeared for IR pullets to have shorter top beak lengths compared to C pullets at 1 day of age. Similar findings have been reported by Henderson et al. [13] and Struthers [14] and suggest that the infrared treatment was already affecting the beak tissue at a cellular level within the first day post treatment. Overall, infrared beak treatment was effective at inhibiting post-treatment beak growth in the present study. This is important as it reduces the chances of (1) having to subject birds to a second, potentially more stressful beak trim at an older age (which would require a different methodology, as infrared beak treatment can only be used at day of hatch) and (2) the beak re-growing enough that birds can successfully cannibalize [7]. Differences between the top and bottom beak lengths remained very small throughout the 21-day period. This indicates that the beak shape was “flush” and that the bottom did not extend out beyond the top creating a step or shovel beak. It has been suggested that any detectable elongation of the bottom beak could be considered a “severe abnormality” and may negatively impact welfare [15]. However, pullets that were infrared beak treated to purposely create a shovel or step beak, production and welfare were minimally affected [16,17]. 

One of the advantages of infrared beak treatment is that it does not result in the immediate loss of the beak tip. Figure 6 demonstrates that prior to the necrotic beak tip sloughing off, the epithelium has regenerated to create a barrier between the newly healed beak tissue and the necrotic beak tip. This lack of an open wound post treatment is important as it reduces the chances of infection once the beak tip sloughs off. By 21 days of age, beaks showed complete healing as evident by the beak being covered in a continuous layer of epithelium. A similar healing response has been observed in birds that were hot-blade trimmed at 1 day or 10 days of age [3]. In both the present study and the one conducted by Gentle et al. [3], the treated beak tips were anatomically normal at 21 days; however, nerve endings and sensory receptors were absent. It is important to reiterate that the difference between the present study and the one by Gentle et al. [3] is that, with infrared beak treatment, the tissue remains to block the opportunity for bacterial infection. The beak samples in the present study were not immunostained for myelin so small nerve fibres may have been present at 21 days of age but the staining technique that was used (H&E) did not allow for them to be observed. The H&E staining technique only allows for the visualisation of different tissues types and inflammatory responses [18]. Future research in this area could focus on using immunohistochemistry and silver staining to identify nerve fibres and examine the effect of infrared beak treatment on them. It is also possible that if the present study had continued for a longer period of time, repopulation of small nerve fibres may have been observed. Prior research has found that birds that were hot-blade trimmed still did not show re-innervation of the beak tip 6 weeks post treatment, whereas birds treated using infrared beak treatment showed some repopulation of sensory receptors and re-innervation in the beak tip as soon as 4 weeks post treatment [3,11]. In both of these studies, neuromas were not present post treatment [3,11]. McKeegan and Philbey [11] concluded that infrared beak treatment did not result in chronic pain due to no effect on nociceptor (pain) thresholds and no neuroma formation observed. However, the authors did not address acute pain. A more recent study conducted by Struthers et al. [16] studied the effect of infrared beak treatment on the pecking force of pullets during early life. They found no differences in pecking force between treated and untreated pullets suggesting that the pullets were not experiencing pain in the beak tissue post treatment [16]. 

## 5. Conclusions

The infrared beak treatment process worked as expected with treated birds having shorter beak lengths and less post-treatment beak growth once sloughing of the necrotic beak tip was initiated. Epithelial regeneration began prior to the initiation of beak sloughing, which reduces the chance that birds will have open wounds at the end of the beak once sloughing is complete. There was no evidence of neuroma formation or abnormal nerve growth, both of which are associated with chronic pain. Overall, the results of this study support the continued use of infrared beak treatment to help improve laying hen welfare and reduce cannibalism until effective and reliable alternatives to beak treatment are found. 

## Figures and Tables

**Table 1:** Infrared beak treatment settings applied to Lohmann Brown-Lite and Lohmann LSL-Lite chicks.

Strain ^1^	Beak Treatment ^2^	Guard-Plate	Mirror	Power
LB	IR	26/23	Aluminum mid-wrap	42
	C	26/23	-	-
LW	IR	26/23	Aluminum mid-wrap	40
	C	26/23	-	-

^1^ LB = Lohmann Brown-Lite, LW = Lohmann LSL-Lite; ^2^ IR = infrared beak treated, C = sham untreated control.

**Table 2:** Beak lesion classifications ^1^.

Score	Description
0	No lesions, triangular shaped beak tip, numerous Herbst corpuscles and mature collagen bundles
0.5	Completely healed epithelium, entire beak covered with epithelium, minimal fibrovascular tissue, triangular- or square-shaped beak tip
1	Completely healed epithelium, entire beak covered with epithelium, minimal inflammation or necrosis, mild to moderate fibrovascular tissue, square-shaped beak tip
1.5	Moderately healed epithelium which is about to unite at beak tip, mild inflammation, moderate fibrovascular tissue, mild to moderate necrotic debris at beak tip
2	Mild to moderate healing, regenerating epithelium covering 50% or more of the beak tissue, moderate inflammation and fibrovascular tissue, moderate necrotic debris and bacterial colonies
2.5	Mild healing, epithelial regeneration is visible as a single cell layer stretched out from the normal epithelium, increased fibroblasts, moderate to severe inflammation and necrosis of the beak
3	Severe coagulative necrosis, hemorrhage, edema, cavitation, severe inflammation, and possible bacterial infection
4	Severe coagulative necrosis, hemorrhage, edema, cavitation of the beak below the treatment line

^1^ Classifications were developed in conjunction with the co-authors of this study (poultry pathologists at the Western College of Veterinary Medicine).

**Table 3:** Effect of infrared beak treatment and strain on the top and bottom beak length and overall beak growth (mm) of Lohmann Brown-Lite and Lohmann LSL-Lite pullets housed in cages from 0 to 21 days of age.

Age (Days)	Beak Treatment ^1^			Strain ^2^			Interaction	SEM
	IR	C	*p*-Value	LB	LW	*p*-Value	*p*-Value	
**Top beak**								
1	5.44	5.53	0.096	5.55 ^a^	5.41 ^b^	0.015	0.759	0.029
7	6.69	6.66	0.605	6.60 ^b^	6.75 ^a^	0.017	0.124	0.030
14	6.91 ^b^	8.54 ^a^	<0.001	7.91	7.53	0.107	0.047	0.162
21	6.06 ^b^	9.91 ^a^	<0.001	7.90	8.08	0.526	0.895	0.512
Growth	0.63 ^b^	4.38 ^a^	<0.001	2.35 ^b^	2.66 ^a^	0.047	0.956	0.533
**Bottom beak**								
1	4.81 ^a^	4.52 ^b^	<0.001	4.79 ^a^	4.53 ^b^	<0.001	0.853	0.037
7	5.92 ^a^	5.47 ^b^	<0.001	5.74	5.66	0.340	0.828	0.044
14	5.82 ^b^	7.02 ^a^	<0.001	6.42	6.40	0.856	0.382	0.135
21	5.57 ^b^	8.40 ^a^	<0.001	6.93	7.04	0.692	0.467	0.385
Growth	0.76 ^b^	3.88 ^a^	<0.001	2.15 ^b^	2.51 ^a^	0.045	0.098	0.449

^a,b^ Means within a main effect with different superscripts are significantly different (*p* ≤ 0.05); ^1^ IR = infrared beak treated, C = sham untreated control; ^2^ LB = Lohmann Brown-Lite; LW = Lohmann LSL-Lite.
